# Fixation Biases towards the Index Finger in Almost-Natural Grasping

**DOI:** 10.1371/journal.pone.0146864

**Published:** 2016-01-14

**Authors:** Dimitris Voudouris, Jeroen B. J. Smeets, Eli Brenner

**Affiliations:** 1 Department of Human Movement Sciences, VU University Amsterdam, Amsterdam, The Netherlands; 2 Department of Psychology, Justus-Liebig University Giessen, Giessen, Germany; Università di Trento, ITALY

## Abstract

We use visual information to guide our grasping movements. When grasping an object with a precision grip, the two digits need to reach two different positions more or less simultaneously, but the eyes can only be directed to one position at a time. Several studies that have examined eye movements in grasping have found that people tend to direct their gaze near where their index finger will contact the object. Here we aimed at better understanding why people do so by asking participants to lift an object off a horizontal surface. They were to grasp the object with a precision grip while movements of their hand, eye and head were recorded. We confirmed that people tend to look closer to positions that a digit needs to reach more accurately. Moreover, we show that where they look as they reach for the object depends on where they were looking before, presumably because they try to minimize the time during which the eyes are moving so fast that no new visual information is acquired. Most importantly, we confirmed that people have a bias to direct gaze towards the index finger’s contact point rather than towards that of the thumb. In our study, this cannot be explained by the index finger contacting the object before the thumb. Instead, it appears to be because the index finger moves to a position that is hidden behind the object that is grasped, probably making this the place at which one is most likely to encounter unexpected problems that would benefit from visual guidance. However, this cannot explain the bias that was found in previous studies, where neither contact point was hidden, so it cannot be the only explanation for the bias.

## Introduction

Visual information guides most of our everyday actions. Human gaze behaviour has been examined in many such actions, such as making tea and sandwiches [1,2], driving a car [1,3], walking [4,5], or engaging in sports [6–9]. Humans typically shift their gaze to positions of interest in anticipation of acquiring information that will help them plan and guide their upcoming movements [10,11]. When hand movements are involved, gaze shifts towards the object of interest before the hand reaches the target [10,12]. Shifting gaze to positions of interest helps people to localize the intended movement endpoints more accurately. Thus, when simultaneously moving the two hands to two different targets, right-handed people sometimes fixate the two targets sequentially, with the fixation just before contact either being near the smaller of the two targets or near the one that will be reached with their left hand [13]. Presumably, gaze is directed towards the position that is expected to provide the most useful information for performing the task.

When grasping an object, people need to first select suitable contact points on the object and then bring their digits to these points [14–16]. When people use a precision grip, the two digits need to arrive at the two positions more or less simultaneously. Studies that examined gaze during grasping while both digits’ contact points were visible found that people tend to initially fixate either close to the anticipated contact point of their index finger or near the object’s centre of mass [17–20]. Only rarely, when higher accuracy was demanded for the placement of the thumb, did people’s gaze shift slightly from the initial fixation near their index finger’s contact point towards their thumb’s contact point [18]. This finding suggests that the fixations depend to some extent on the required accuracy of each digit’s placement on the object. However, this does not explain why people show a strong preference to fixate near the anticipated contact point of their index finger, because the variability of the index finger’s contact points is not systematically larger than that of the thumb [21,22].

The index finger generally moves along a more curved trajectory towards its contact point [22–25]. To examine whether this characteristic of the index finger’s trajectory could be responsible for the bias of looking towards the index finger’s upcoming contact point, Cavina-Pratesi and Hesse (2013) conducted a study in which grasping movements started at two positions, one for which the thumb had to curve around the object in order to reach its contact point, and the other for which the index finger had to do so. Despite the more curved trajectory of the thumb than of the index finger for one of the initial hand positions, participants always fixated the position at which the index finger would contact the object. It was proposed that the bias of looking towards the index finger’s contact point might be related to the index finger generally contacting the object earlier than the thumb [19]. In accordance with this proposal, people tend to look closer to their thumb’s contact point when the thumb is the first to make contact with the object, which is the case if the object that is to be grasped is at eye height so that the index finger’s contact point is behind the object, well out of sight [26]. However, there did not appear to be any correlation between which digit first touched the object and where people directed their gaze in an earlier study in which both contact points were visible [18].

Altogether, most studies find that people direct their eyes close to the positions at which their index finger will contact the object, but none of them has managed to explain this behaviour. In most studies that recorded eye movements during grasping, participants grasped a thin target object that was attached to a frontal screen, mostly by placing their finger at the top and the thumb at the bottom of the object [17–19]. In such cases, manipulating the object without dropping it might give the index finger a different role than the thumb. For instance, the way the object is removed from such a frontal surface might result in a need to fixate near the index finger’s contact point. In some more natural grasping situations people look closer to their thumb’s contact point [10,26], perhaps because this is the only visible contact point since the index finger’s contact point is behind the object, and therefore occluded by the object itself. We therefore decided to re-examine where people look when grasping under natural grasping conditions. We placed the target object on a horizontal surface, below eye height but close to the participant, without restricting head movements so that participants could bend forward to look down at the object from above. When picking up an object off a horizontal surface, the constraints on the placement of the digits may still differ, but at least the constraints are more similar to those routinely experienced in daily life. We also examined how gaze changed when we manipulated various constraints.

In a series of seven experimental sessions, we asked participants to grasp either a block or a sphere. The object was placed on a table in front of them. The participant’s head was not constrained (unlike in earlier experiments; [17–20]). The participants were standing right in front of the table, and the object was close to the near edge of the table, so that the participants had to look down to see the object. Consequently, the digits’ contact points were about orthogonal to the line of sight and both digits’ trajectories towards their contact points were always visible. We used this configuration because if participants had been sitting at a table, reaching for an object that was some distance away at chest or eye height, the index finger would primarily be further away than the thumb at the moment that the object was grasped [10,27]. In that case the index finger would require a similar orientation of the eyes to be fixated as the thumb, but would be hidden behind the object. The rationale of the details of each experiment is combined with the presentation of details that are specific to that experiment when introducing the separate experiments.

## Methods

We performed seven experiments, each in a separate session. Since the experiments were all very similar to each other, and the changes that we made were guided by the results of the preceding experiment, we describe the general methods here, and the precise conditions when introducing each of the experiments.

### Participants

In total, fourteen healthy participants took part in the experiments. Seven or eight participants took part in each experimental session, so not all participants took part in all sessions. We numbered the participants from P1 for the youngest (26 years old) to P14 for the oldest (32 years old), with P1, P10, P13 and P14 being male, and the other 10 female. The participants were unaware of the purpose of the study and gave their signed informed consent prior to the experiment. All were right-handed by self-report and had normal vision. This study was part of a program that has been approved by the ethical committee of the Department of Human Movement Sciences of the VU University Amsterdam.

### Apparatus and experimental setup

**Fig 1:**
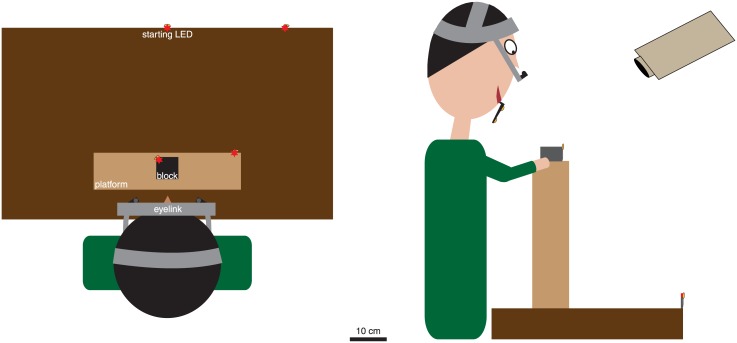
Top and side views of the setup (partially drawn to scale). The top view shows the markers that were relevant during the calibration of the eye movements. Participants switched their gaze between four LEDs (red stars) that were turned on sequentially in response to the previous LED being fixated. The LEDs’ positions with respect to the eyes were determined by measuring the positions of infrared markers (yellow shapes) next to each LED and on the bite-board (not visible; see side view). The positions of the eyes were determined from the positions of the markers on the bite-board. The side view shows the markers and LEDs that were used during most sessions. Additional markers were attached to the index finger and thumb. We ensured that the markers on the object, index finger and thumb would not interfere with the movements. The distance to the Optotrak was much larger than is shown.

The position and orientation of the participant’s head, and the positions of the thumb and index finger of his or her right hand, of the target object, and of a fixed point in space were all recorded at 100 Hz (resolution of about 0.1 mm) with an Optotrak 3020 motion tracking system (Northern Digital, Waterloo, ON, Canada). Three infrared markers were attached to an individually fitted dental-impression bite-board that the participant held in his or her mouth, but that did not restrain his or her movements. The positions of these three markers were used to determine the position and orientation of the participant’s head. Single infrared markers were attached to the nails of the thumb and index finger of the participant’s right hand. An additional single infrared marker was attached either to the far-left of the target block’s upper surface (see also Fig 1) or to the leftmost position of the target sphere (as seen by the participant), depending on the experiment. A final marker was aligned with the *starting* LED (a red light emitting diode indicating the starting position of the participant’s gaze). The starting LED was at the far edge of the table (as seen by the participant; Fig 1), except in experiment 2, in which it was at the near edge of the table. This marker was approximately aligned with the participant’s midline. Eye-in-head movements of both eyes were recorded at 500 Hz (resolution of about 0.2°) with an Eyelink II eye-tracker (SR Research Ltd.). For the calibration of the eye movement recordings (see *Determining gaze trajectories* below) three additional light-emitting diodes (LEDs) were used, each aligned with an infrared marker along the participant’s line of sight (as shown in Fig 1).

The experiment was performed in a normally illuminated room. The participant stood in front of a wooden table (90 cm wide; 52 cm deep; adjustable height; Fig 1), on top of which a wooden platform (40 cm wide; 10 cm deep; 40 cm tall) was placed. Depending on the experiment, the target object was either a wooden block (top surface of 6 x 6 cm; height of 4 cm; 110 g) or a sphere made of foam (6.7 cm diameter; 14.1 g). The target object was placed on the wooden platform, about 15 cm from the participant’s trunk. It was approximately aligned with his or her midline. The initial position of the hand depended on the experiment, and never coincided with the starting LED.

### Determining gaze trajectories

**Fig 2:**
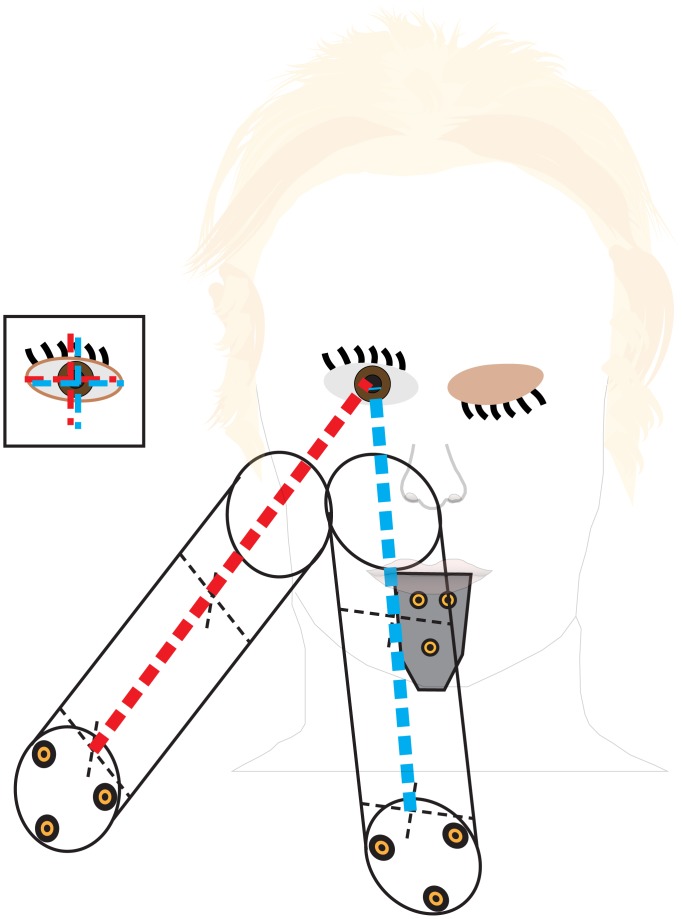
Determining the relation between eye and bite-board position. Two possible orientations of the tube for which the two crosses (the thin dashed black lines) are visually aligned for the participant’s right eye (see inset). The thick dashed lines cross each other at the position of the eye. By measuring the orientations of the tube with respect to the bite-board (shown in grey), we determine the 3D position of the eye relative to the bite-board.

In order to combine head orientation data as measured using the bite-board with the eye-in-head data of the eye-tracker, we had to calibrate the set-up. The first step in the calibration was to relate the Cartesian positions of the participant’s eyes to the positions of the markers on the bite-board. This was done once for every participant, in a separate session before the actual experiments, using a method that was introduced before [28]. The participant looked with one eye through a long tube (length: 50 cm; diameter: 5 cm) while holding the bite-board in his or her mouth (Fig 2). There were three infrared markers at fixed positions on the tube’s far end (relative to the participant). Participants saw two crosses, each formed by a pair of orthogonal threads that intersected each other on the main axis of the tube. One cross was halfway down the tube and the other at the tube’s far end.

The participant was asked to look through the tube with one eye and align the two crosses. This could be done by moving the head, moving the tube, or a combination of the two. Once the two crosses appeared to the participant to be aligned, the participant pressed a key in response to which the positions of all the markers were registered (averaged over the preceding 100 ms) unless any of the markers was moving (standard deviation in position of more than 1 mm during the preceding 100 ms), in which case a sound was heard and the alignment had to be repeated. The two crosses only looked aligned when the eye was situated along the tube’s main axis, so each measurement constrained the Cartesian position of the eye to a line, whose location is known relative to the bite-board.

The crosses were aligned 25 times, while ensuring that the orientation of the tube varied with respect to the participant’s head, so that the lines would fan out from the eye in many directions. The point relative to the bite-board for which the sum of the squared distances to the 25 lines was the smallest was considered to be the centre of the eye (after removing outliers; lines that shifted the point by more than 1 mm). Knowing this point with respect to the position of three markers on the bite-board, we could tell where the participant’s eye was, as long as he or she held the bite-board in his or her mouth (and the markers were visible). This calibration was performed separately for each eye. It took approximately 5 minutes per eye.

The second step in the calibration was to relate the raw output of the eye-tracker to orientations of the eye in the head. This was done at the beginning of each experimental session. The raw eye tracker values correspond with the position of the pupil as seen by the camera. The eye-tracker was attached to the participant’s head and the bite-board was placed in their mouth. Four LEDs, each accompanied by an infrared marker, were placed at strategic positions in the workspace (Fig 1). Initially, one of the four LEDs emitted light. The participant was asked to fixate that LED. Once a fixation was detected, the fixated LED was turned off and the next one was turned on. This switch was accompanied by a short tone. The procedure was continued for 6 complete cycles through the 4 LEDs (24 fixations). Participants were asked to try not to move their head during this step of the calibration to ensure that the gaze shifted relative to the head. For each eye, we fit the raw horizontal and vertical values provided by the eye tracker to the corresponding position of the LED relative to the head, to relate raw eye tracker values to eye-in-head angles. In doing so, we considered all 24 fixations, except ones for which the raw eye tracker values differed by more than 2.5 standard deviations from the average raw eye tracker value for that LED.

Based on the gaze-directions of both eyes, we could in principle determine a 3D gaze-position in space. However, as the depth-component (based on vergence) is very imprecise, and the positions of the finger and thumb when grasping the objects were more or less at the same distance from the participant, we decided to present 2D gaze-directions, obtained by averaging the data of the two eyes. We used the direction of the line connecting the two eyes as the horizontal direction (which is relevant, as the head might tilt). This is the most natural coordinate system for reporting eye movements. In order to relate the gaze-patterns to the grasping behaviour, we use the same angular coordinate system to represent digit trajectories. It is important to realize that since participants were free to move their heads, fixed positions in space, such as the starting LED and the initial position of the hand, varied slightly in position in this representation.

### Procedure

Participants stood in front of the wooden table. The table’s height was aligned with the participant’s hip. The eye-tracker’s cameras were positioned so that they did not occlude any parts of the workspace. After calibrating the eye movements, the experimenter removed all the LEDs except the starting LED, and attached single infrared markers to the nails of the participant’s right thumb and index finger, taking care that the wires did not interfere with the participant’s arm movements.

Each trial started with the participant bringing his or her hand to the initial hand position for that experimental session and fixating the illuminated starting LED. Meanwhile, the experimenter placed the object at the appropriate position (thus the object was not occluded from the participant). The experimenter then pressed a computer key, which started the recording and switched off the LED to indicate that the participant could start moving. During the trial, the participant could look wherever he or she wanted. The task was to reach and grasp the target object with a precision grip (between thumb and index finger), lift it, put it back down near its original position, and move the hand back to its initial position. The participant then fixated the starting LED in anticipation of the next trial.

No instructions were given as to where participant should look (except when the trial started) or how to grasp the object (other than that it was to be done with a precision grip). Participants always lifted the object within a few seconds. Each of our conditions (that will be described in the introductory sections for each experiment) was presented 22 times, and there were either two or three conditions in each experimental session, so each session consisted of 44 or 66 trials, that were presented in a pseudo-random order.

### Data analysis

A number of measures were only determined in order to obtain other measures, such as when the grasping movement started and when each of the digits made contact with the object. The measures that we will analyse are printed in *italics*. We determined the velocity of the hand by numerical differentiation of the average of the positions of the markers attached to the thumb and index finger. A velocity threshold of 10 cm/sec determined the onset of the hand movement. Grip aperture was defined as the three-dimensional distance between the markers on the two digits.

The moment of contact was determined for each digit separately using the Multiple Sources of Information method [29]. The moment of contact was after maximal grip aperture and not more than 60 ms before the object first started to move (i.e. before the position of the marker on the object differed by more than 3 standard deviations from its average position for the period up to the moment of maximal grip aperture). The likelihood of a moment being the moment of each digit’s contact increased in proportion to the absolute value of the acceleration of that digit (there was a clear deceleration peak due to the contact itself). The likelihood also increased linearly with the extent to which the distance between the digit and the marker on the object was similar to their distance when the object was being lifted; it was 1 when the distances were identical and 0 when the difference in distance was maximal (when the digits were near their initial positions). For each digit, the moment at which the product of these likelihoods was largest was considered to be the moment that the digit contacted the object. If two consecutive samples had a product that differed by less than 5%, the first of them was considered to be the moment of contact. If there were two non-consecutive samples at which this product differed by less than 5%, the trial was rejected (due to uncertainty about the moment of contact). Likewise, if the moments of contact of the two digits differed by more than 200 ms, or if the marker on the other digit or on the object were occluded around the moment that a digit made contact, the trial was rejected.

The digit that contacted the object first was the one on which we based the moment of contact when analysing gaze. *Contact asynchrony* was the time difference between the two digits’ moments of contact, with positive values indicating that the index finger contacted the object earlier than the thumb.

Grip orientation was used to determine by which surfaces the block was grasped and the angular grip size was determined to be able to report its approximate magnitude for comparison with the gaze data. The final grip orientation was defined as the angle of the projection on the horizontal plane of a line connecting the two markers on the digits at the moment of the grasp. *Final grip size* was the length of that line, and is expressed as a visual angle. Looking at plots of the digits’ trajectories (available in the results sections) confirmed that final grip orientation provides reliable information about the surfaces by which the objects were grasped, despite being based on the positions of the markers and not on the actual positions of the fingertips that made contact with the object. Final grip size provides a measure for relating magnitudes of shifts in gaze to the angular size of the object. This measure slightly overestimates the distance between the contact points because it too is based on the positions of the markers rather than the fingertips.

The first step in the analysis of the eye movements was to convert the raw data from the eye-tracker to gaze trajectories expressed in visual angles on the basis of the calibration. We used the measured positions of the markers on the bite-board to determine the Cartesian positions of the eyes and to identify the horizontal direction. To determine the direction of gaze, we had to combine data from the eye-tracker with Cartesian positions that were sampled at a lower frequency. We did so by selecting the eye-tracker value that was the closest in time for each sample of the Cartesian positions of the eyes, resulting in two 100 Hz gaze trajectories (one for each eye) that were then averaged. We next converted the positions of all relevant landmarks (including the positions of the digits) to the same coordinate system. We could hereby express distances between gaze and any relevant landmarks as gaze angles. We corrected for drifts of the Eyelink system by assuming that the gaze is at the starting LED when fixating the latter at the onset of each trial (changes in the median of 5 trials were considered to be caused by drift; e.g. due to the Eyelink’s headband slipping).

In order to separate fixations from saccades, gaze angular velocity was calculated by numerical differentiation of the gaze trajectory. The onset of a saccade was the first sample on which the gaze velocity exceeded a threshold of 35°/sec. The onset of a fixation was the first sample on which the velocity dropped back below 35°/sec. We were mainly interested in the critical fixation, which is the gaze when the first digit made contact with the object, or just before the saccade if a saccade was being made at the moment of contact. We also determined the first fixation, which was where participants fixated after their first saccade away from the starting LED. We determined the visual angle between each of these two fixation points and each of the two contact points. The difference between the two visual angles for each fixation point was taken as a measure of the extent to which gaze was closer to the index finger than to the thumb. We refer to this difference as the *initial gaze bias* when considering the first fixation and as the *critical gaze bias* when considering the critical fixation. In both cases, positive values indicate that fixation is closer to the index finger.

To examine the extent to which gaze changed during the reach-to-grasp movement, and thus whether participants regularly shifted their gaze between the contact points, we determined the *maximal distance between any two fixations* (in degrees of visual angle) from the first fixation until the critical fixation. Note that we determine the maximal distance between any two fixations, not the maximal distance between consecutive fixations, so this does not require a single saccade to have been made between these positions. We determined this maximal distance for each trial, and report the median value across trials. We report the median rather than the mean because the distances are not normally distributed: they can only be positive and they are sometimes exceptionally large because participants occasionally shift their gaze back to the starting LED, presumably in anticipation of the next trial, before touching the object. If the *median maximal distance between any two fixations* is much smaller than the final grip size, participants cannot have regularly shifted their gaze between the contact points.

Except for discarding trials because we were uncertain about the moments of contact, we also discarded trials if gaze was missing for more than half the duration of the reach-to-grasp movement (e.g. due to equipment failure), if gaze data was missing during the last 200 ms before the grasp (e.g. due to blinks), or if the participant did not fixate the starting LED at the beginning of the trial. We determined the initial and the critical gaze biases, the contact asynchrony, the final grip size and the maximal distance between fixations for each trial. We then determined the average (or the median for the maximal distance between fixations) across the repetitions for each condition performed by each participant. The influence of the different conditions on the critical gaze bias and contact asynchrony was evaluated with one-way repeated measures ANOVAs. The consistency of gaze biases (deviation from zero) was evaluated with one-sample t-tests. To evaluate the possible relationship between contact asynchrony and critical gaze bias, we determined the correlation between the participants’ average contact asynchrony and their average critical gaze bias for each experimental session.

## Experiment 1—Selecting Sides

In our first experiment, we attempted to replicate the finding that people fixate near their index finger when grasping an object, but using a more natural configuration than has been studied until now. Participants were asked to reach and grasp a block that was placed in front of them. The initial hand position was at the right edge of the platform as seen by the participant (approximately 20 cm from the object). The block could have one of three different orientations (45°, 25° or 65° relative to the participant’s frontal plane; we will refer to these orientations as *middle*, *clockwise* and *anti-clockwise*, respectively). We used several orientations (three conditions) because previous studies [30,31] showed that some participants switch between grip orientations, for the same object orientation, when it is not clear which pair of surfaces is best for grasping the object. Them doing so would allow us to distinguish between looking near where the index finger will make contact with the object, and looking at the upper part of the object (in terms of the visual image) for some other reason. In the former case, gaze should follow the index finger if the index finger moves to a different surface. In the latter case it should not, because the visual image is the same. More generally, if participants direct their gaze towards one of their contact points, we expect their critical fixations, for a given object orientation, to change if their grip orientation changes.

### Participants

Eight participants (P1, P3, P7—P12) took part in this experiment. Of the 528 trials (8 participants; 3 block orientations; 22 replications), 66 had to be discarded, mainly because markers on the digits were occluded and because of uncertainty about the contact moments, resulting in 462 trials for further analysis (the number of remaining trials per condition was similar to that in previous studies [17–20]).

### Results

**Fig 3:**
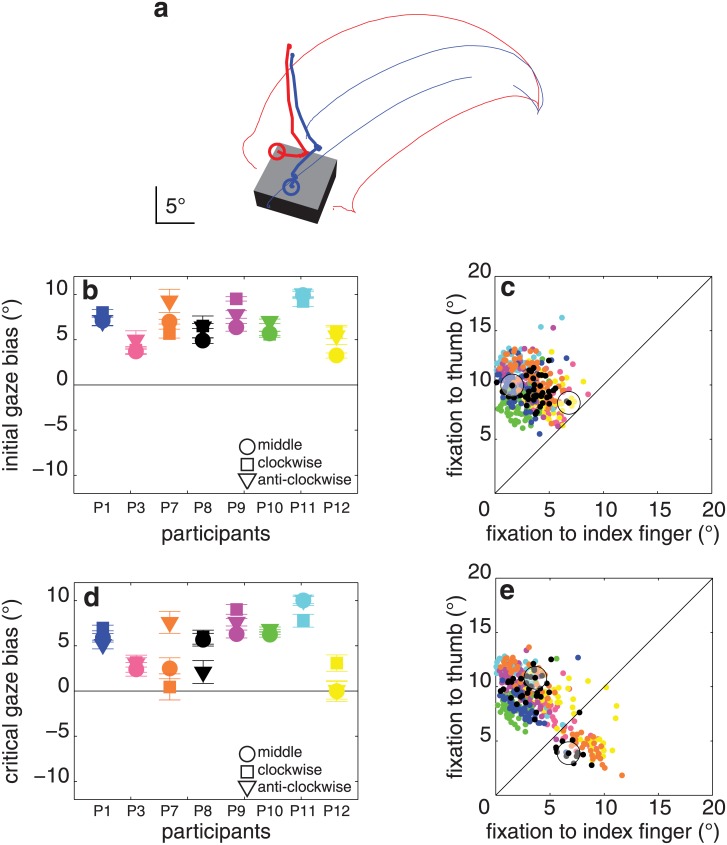
Results of experiment 1. (**a**) Two trials of a single participant (P8) for one object orientation (blue and red lines). Trials were selected for which the object was grasped by different pairs of surfaces. Thin lines: digits’ trajectories (the thumb’s marker was briefly missing, early during one of the grasping movements). Thick lines: gaze trajectories. Circles: gaze at the moment the first digit touches the object. The block is drawn as this participant saw it at the first moment of contact (averaged across all trials in which it had this orientation). (**b**) Initial gaze bias (how much closer the first fixation is to the index finger’s than to the thumb’s contact point) for each block orientation and participant (with standard errors across replications). Each colour represents one participant. Negative values would mean that the fixation is closer to the thumb’s contact point. **(c)** Distance between the first fixation and each of the two contact points for each of the 462 trials. Colour coding as in **b**. The trials within the circles are the trials shown in panel a. (**d, e**) Same as **b**, **c** for the critical fixation.

Fig 3a shows two trials of a single participant for the middle object orientation. In one of these trials the participant first moved her gaze down to near where the index finger would make contact, and subsequently to near where the thumb would do so (blue thick trajectory). In the other trial she first moved her gaze down to near where the index finger would have made contact if the object were grasped in the same manner as in the previous example, and subsequently to near where her index finger actually made contact (red thick trajectory).

The participants showed a large initial gaze bias: 6.7 ± 1.8° (average ± standard deviation between participants, t_7_ = 10.5, p < 0.001; Fig 3b and 3c). The critical gaze bias was slightly smaller than the initial gaze bias: 5.1 ± 2.9° (t_7_ = 5.1, p < 0.001; Fig 3d and 3e), and was independent of the object’s orientation (F_2,14_ = 0.4, p = 0.7; Fig 3d). The contact asynchrony was 21 ± 11 ms, and was independent of object orientation (F_2,14_ = 1.3, p = 0.3). There was no significant correlation between contact asynchrony and critical gaze bias (r = 0.59, p = 0.1). Participants did not systematically look back and forth between their contact points during the reaching movements. The median maximal distance between any fixations that took place during individual reach-to-grasp movements was 3.6 ± 2.5°. The average final grip size was 10.8 ± 1.2°.

**Fig 4:**
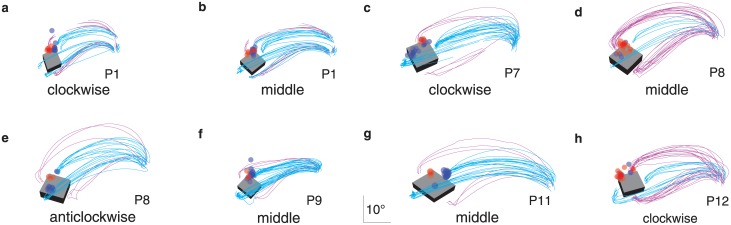
Digits’ trajectories and critical fixation points when participants grasped the same block by different pairs of surfaces in experiment 1. All the trials of all combinations of participant and block orientation for which the block was grasped by each of the possible pairs of surfaces at least three times. Each panel shows the data of one combination of participant and block orientation. Cyan and purple lines represent the digits’ trajectories when grasping by the two pairs of surfaces. Blue and red dots represent the critical fixations when grasping by the two pairs of surfaces. Note how the (average) image of the block (at the time of contact) and the scaling of each image are different for the different participants. This is because the image depends on the positions of the participants’ eyes, which depend on how tall the participants are and how they choose to stand, as well as on the orientation of the block. Participants, except for the ones shown in panel **f** and **h**, clearly shifted their critical fixations when they adopted a different grip orientation. The shifts are most pronounced in panels **c**, **e** and **g** in which the two types of grip orientations are clearly separated.

For some orientations of the block, some participants did not always grasp the block by the same two surfaces. When participants grasped the block by a different pair of surfaces than on other trials, they usually also directed their critical fixations closer to those surfaces (red dots closer to magenta trajectories and separated from the blue dots that are closer to the cyan trajectories in Fig 4). Thus, when naturally grasping a block, participants tend to look near where the index finger will make contact with the block, rather than just towards the ‘upper’ part of the block.

### Discussion

In this first experiment, we found that the fixation bias that is reported in the literature [17–20] is not limited to grasping off a frontal surface: people also fixate near where their index finger will make contact with an object when the task is to grasp and lift the object off a horizontal surface. That they were looking at the contact points, rather than at the top of the object, is supported by the shifts in gaze when different grip orientations were adopted (Fig 4). In the subsequent experiments we will examine several possible reasons for looking closer to the index finger’s contact point than to the thumb’s contact point in this experimental configuration (with the object at eye height gaze appears to be directed towards the thumb’s contact point [26]). Not finding a significant correlation between contact asynchrony and critical gaze bias is inconsistent with the idea that gaze is related to which digit is the first to make contact with the object [19,26].

## Experiment 2—Reducing Saccade Amplitude

In the first experiment, we found that participants usually initially shifted their gaze to near their index finger’s contact point. They then sometimes shifted it to other positions. A question that arises is whether the initial fixation was really to the position at which the index finger would make contact. Participants might prefer the shortest eye movement with which they could fixate a future contact point. For the configuration of experiment 1, doing so would direct the participants’ gaze to where their index finger will make contact with the object. Making as small a saccade as possible could help obtain the necessary information as soon as possible. If participants prefer small saccades, one would expect the initial fixation to be near their thumb if the starting LED is placed between the participant and the object. The second experiment was therefore identical to the first, with the only difference that the starting LED was placed between the participant’s midline and the object, at the close edge of the wooden table (relative to the participant). As a result, participants had to shift their gaze upwards to fixate the target object, instead of downwards as in the first experiment.

### Participants

Seven participants (P1, P4—P6, P9—P11) took part in this experiment. Of the 462 trials (7 participants; 3 block orientations; 22 replications), 40 had to be discarded, mainly because markers on the digits were occluded and because of uncertainty about the contact times, resulting in 422 trials for further analysis.

### Results

**Fig 5:**
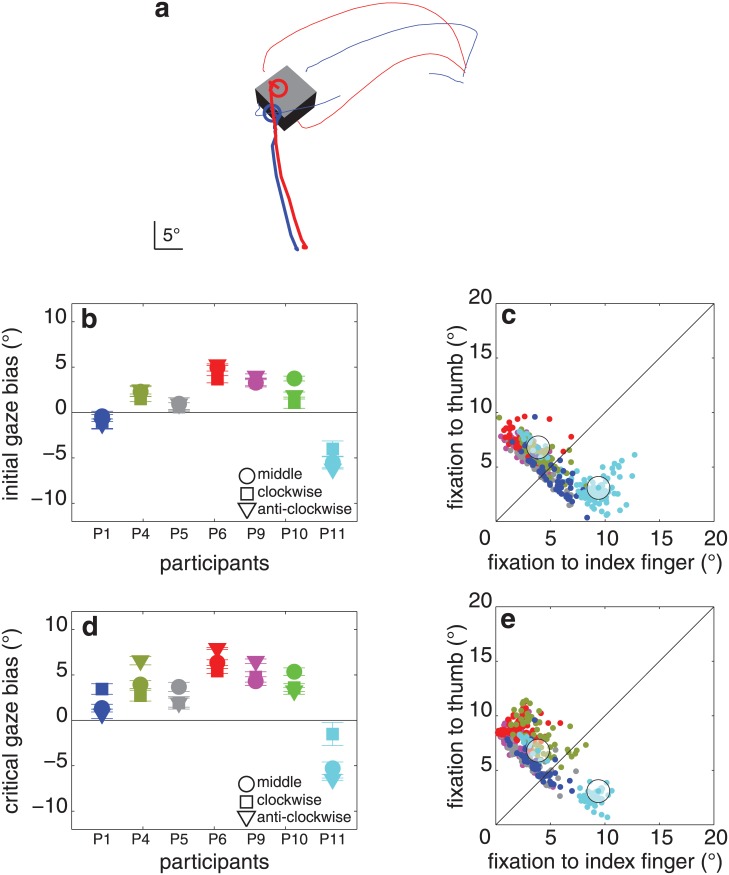
Results of experiment 2. (**a**) Two trials of a single participant (P11) for an object orientation for which the object was grasped by different pairs of surfaces (blue and red lines). (**b**) Initial gaze bias, for each block orientation and participant. **(c)** Distance between the first fixation and each of the two contact points for each of the 422 trials. (**d, e**) Same as **b**, **c** for the critical fixation. Details as in Fig 3.

Fig 5a shows two trials of a single participant for the middle object orientation. Again, we selected to show two trials in which the object was grasped by different pairs of surfaces. In one of these trials the participant fixated close to where the thumb made contact with the object (blue thick trajectory). In the other trial she brought her gaze somewhere in between the object’s centre and where her index finger made contact with the object (red thick trajectory).

Starting with one’s gaze closer to the thumb’s anticipated contact point removed the initial gaze bias that we found in the first experiment. The bias was now 0.8 ± 3.1° (t_6_ = 0.8, p = 0.4; Fig 5b and 5c). The critical gaze bias tended to be more positive than the initial bias (2.8 ± 3.5°) but it too was not significantly closer to the index finger’s contact point than to that of the thumb (t_6_ = 2.1, p = 0.07; Fig 5d and 5e). Importantly, only one participant now consistently had a negative critical gaze bias. The other 6 participants still had a positive critical gaze bias. The object’s orientation did not influence the critical gaze bias (F_2,12_ = 0.8, p = 0.9; Fig 5d). There was no effect of object orientation on the contact asynchrony (F_2,12_ = 3.6, p = 0.06), which was 18 ± 5 ms. No significant correlation was found between contact asynchrony and the critical gaze bias (r = -0.45, p = 0.3). The median maximal distance between fixations during a trial was 3.2 ± 1.1°. The average final grip size was 8.9 ± 0.8°.

**Fig 6:**
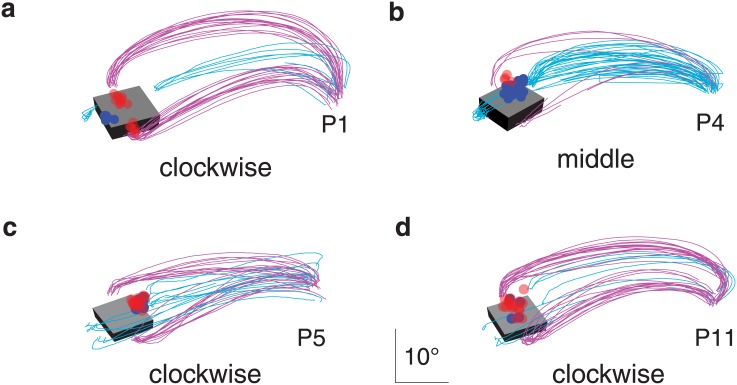
Digits’ trajectories and critical fixation points when participants grasped the same block by different pairs of surfaces in experiment 2. All the trials of all combinations of participant and block orientation for which the block was grasped by each of the possible pairs of surfaces at least three times. Panels **a** and **b** show data for two cases in which gaze clearly shifted with the grip orientation. Panels **c** and **d** show two cases in which it clearly did not. Details as in Fig 4.

The critical fixation only clearly depended on the surfaces by which the object was grasped in two of the four cases for which this could be judged in experiment 2 (Fig 6). The bias certainly did not switch to the thumb (negative values) when gaze started below the object, so although minimizing the initial saccade amplitude may contribute to the fixation biases, it cannot be the main reason for the critical gaze bias towards the index finger’s contact point.

### Discussion

Comparing the results of the first two experiments, we see that our participants’ first fixations on the object were lower in the visual field, closer to the thumb’s contact point, when gaze started closer to the body (experiment 2) than when gaze started at the far edge of the table (closer to the index finger’s contact point, experiment 1). This is probably the result of a tendency of saccades to undershoot the aiming location (that might have been the same for both experiments). Saccadic undershoot could help obtain visual information sooner, because the larger the amplitude of the saccade, the longer the eye will be moving so fast that no useful new information can be obtained [32]. However, despite the fact that changing the initial gaze orientation changed the fixation positions, only one participant had a negative gaze bias, fixating near her thumb’s contact point, when the gaze started closer to the body. Since most average initial gaze biases were positive in experiment 2, despite the eyes always being directed closer to the thumb’s contact point before the first saccade, and since the critical gaze biases were more positive, it is evident that making small saccades is not the only reason for systematically fixating near the index finger’s contact point.

## Experiment 3—Earliest Arriving Digit

Comparing experiments 1 and 2 we see that placing the starting LED between the participant and the object, rather than behind the object, reduced the initial gaze bias from 6.7° to 0.8°, and the critical gaze bias from 5.1° to 2.8°. Thus, reducing the saccade amplitude and duration to quickly obtain visual information about the target probably contributes to the biases. However, even with the starting LED between the participant and the object, most participants’ gaze was biased towards the index finger’s contact point, so reducing the saccade amplitude and duration is clearly not the main reason for people to look closer to the index finger’s contact point.

**Fig 7:**
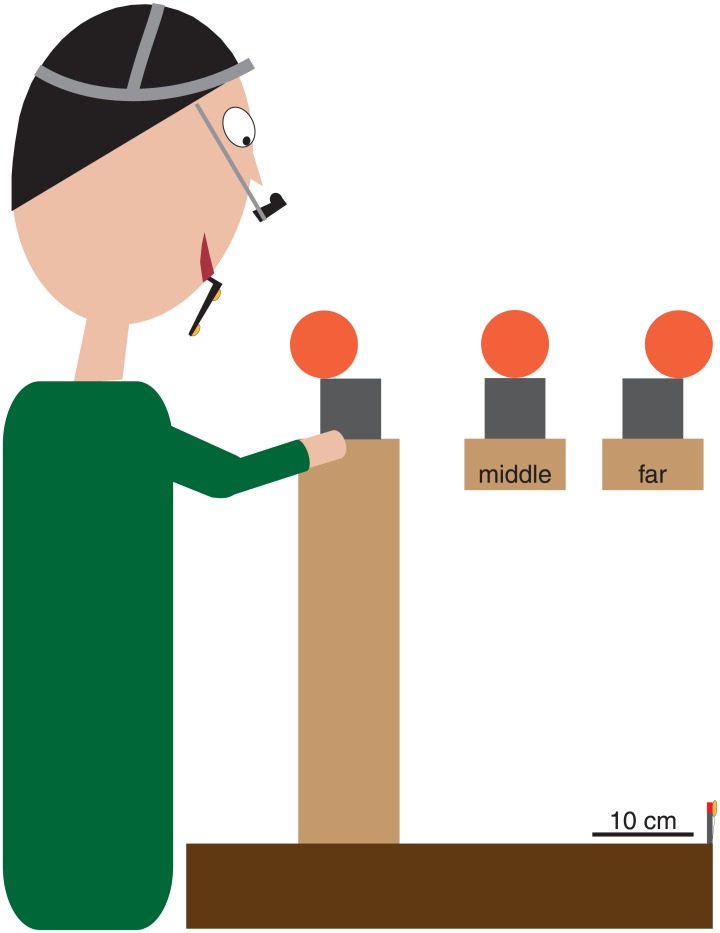
Side view of the setup in experiment 3 (approximately to scale). The sphere was placed at one of three positions on an additional support. When the sphere was carefully placed at the close edge of the support (as shown), there was a danger of pushing it off the additional support with the index finger if the thumb was not there in time. When the sphere was placed at the far edge (see inset), there was a danger of pushing it off with the thumb if the finger was not there in time. When the sphere was at the middle (see inset) there was less danger of accidentally pushing it off the support.

The index finger generally made contact with the object earlier than the thumb (as was already observed by [18,19,30,33]; but see:[26,34]). The lack of significant correlations between gaze bias and contact asynchrony across participants in our first two experiments is consistent with the finding that the tendency to fixate closer to the index finger is not related to which digit makes contact with the object first on individual trials [18]. However, the differences between participants’ biases and between their contact asynchronies may simply have been too small to reveal any correlations. We therefore decided to try to make people clearly want to first make contact either with their thumb or with their index finger, without explicitly asking them to do so. For this purpose, we placed a sphere at three different positions on an additional support (12 cm wide; 6 cm deep; 6 cm tall) that was placed on the platform (Fig 7): a *middle* position, a position at the *far edge* of the support (relative to the participant), and a position at the *close edge* of the support. We asked participants to grasp the sphere without it falling off the support. All other details were as in experiment 1.

We expected the index finger to touch the object slightly earlier than the thumb, as before, when the object was in the *middle position*. We also expected the index finger to touch the object first when the object was at the *far edge*, because doing so reduces the chance of tapping the object off the support with one’s thumb. The most interesting condition was when the object was placed at the *close edge*. In this case, we expected participants to make contact with the object with their thumb before they did so with their index finger (i.e. a negative *contact asynchrony)*, in order to reduce the chance of tapping the object towards oneself, off the support, with one’s index finger.

### Participants

Eight participants (P4, P5, P7, P9—P13) took part in this experiment. Of the 528 trials (8 participants; 3 sphere positions; 22 replications), 41 had to be discarded, mainly because markers on the digits were occluded and because of equipment failure, resulting in 487 trials for further analysis.

### Results

**Fig 8:**
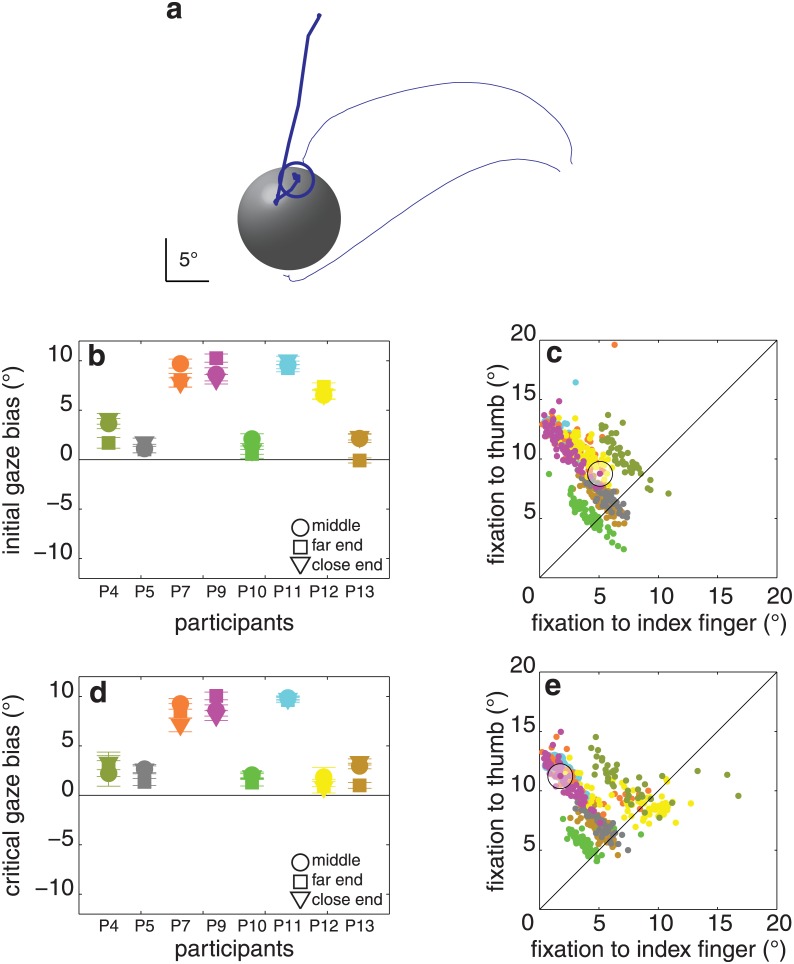
Results of experiment 3. (**a**) One trial of a single participant (P9) for the object at the middle position. (**b**) Initial gaze bias for each sphere position and participant. **(c)** Distance between the first fixation and each of the two contact points for each of the 487 trials. (**d, e**) Same as **b**, **c** for the critical fixation. The clear clustering of individual participants’ data in panels **c** and **d** is due to individual participants’ eyes being at different distances from the object, so that for any fixation position between the two contact points the sum of the distances between fixation and the two digits differs systematically between participants. Details as in Fig 3.

Fig 8a shows one trial of a single participant for the object in the middle position. The participant directed her gaze to near where her index finger made contact with the object (blue thick trajectory).

The sphere never fell off the support. Importantly, the intended effect of the sphere’s position on contact asynchrony was not obtained (F_2,14_ = 0.2, p = 0.7; average asynchrony: 11 ± 19 ms). The initial gaze bias was 5.8 ± 3.1° (t_7_ = 3.9, p < 0.05; Fig 8b and 8c). The critical gaze bias was 4.7 ± 3.4° (t_7_ = 3.6, p < 0.05; Fig 8d and 8e). Given the lack of effect of our manipulation on the contact asynchrony, it is not surprising that the sphere’s position did not influence the critical gaze bias either (F_2,14_ = 0.8, p = 0.4; Fig 8d). No significant correlation was found between the contact asynchrony and the critical gaze bias (r = 0.43, p = 0.2). The median maximal distance between fixations during a trial was 3.1 ± 2.4°. The average final grip size was 12.7 ± 2.3°.

### Discussion

In this experiment we hoped to systematically make our participants first touch the sphere with either their thumb or index finger without explicitly asking them to do so. We expected that placing the sphere near the edge of an additional support would make people touch it with the digit that was at the side of the edge before touching it with the other digit to make sure not to roll it off the edge. Unfortunately, this manipulation failed to influence the contact asynchrony, so we still cannot tell whether increasing the contact asynchrony would reveal a relationship between contact asynchrony and gaze. Nevertheless, this experiment shows that the shape of the target object (block or sphere) is not a critical factor for the gaze bias.

## Experiment 4—Curving Digits

In experiment 3, placing a sphere on the *near edge* of an additional support surface failed to make people contact the object with their thumb before they made contact with their index finger. This can be explained in hindsight by the difference between the timing of contact by the two digits being so small that the other digit was always close enough to the sphere to ensure that it would not roll off the edge. Thus, we could not completely rule out the possibility that differences in the average contact asynchrony are responsible for the bias in eye movements, but the fact that we failed to influence the contact asynchrony suggests that the contact asynchrony is unlikely to arise from evaluating the risks involved in first making contact with each of the digits.

Another possible origin for the biases in fixations is that the index finger generally curves around the object to reach its contact point. This curvature may make reaching this contact point harder, and therefore people may need more visual information about this area. This did not seem to be the reason for the gaze bias when grasping thin objects that were attached to a frontal plane [19], but in that case there was no danger of knocking into the object while passing it, so we decided to examine this for grasping a block off a surface. We compared grasps starting from either a *near* or a *far* initial position of the hand, each about 20 cm from the block at the same height as the top of the platform (*near*: between the participant and the block; *far*: between the block and the starting LED). We oriented the block in a way that ensured that the index finger had to curve around the block when starting *near*, whereas the thumb had to curve around the block when starting *far*. We examined whether people are more likely to look at their thumb’s contact point when starting their grasping movements from the *far* position. Again, all other details were identical to those of experiment 1, except that we only used one orientation of the block (90° relative to the frontal plane).

### Participants

Eight participants (P1, P3, P6, P8—P11, P14) took part in this experiment. Of the 352 trials (8 participants; 2 initial hand positions; 22 replications), 28 had to be discarded, mainly because markers on the digits were occluded and because of equipment failure, resulting in 324 trials for further analysis.

### Results

**Fig 9:**
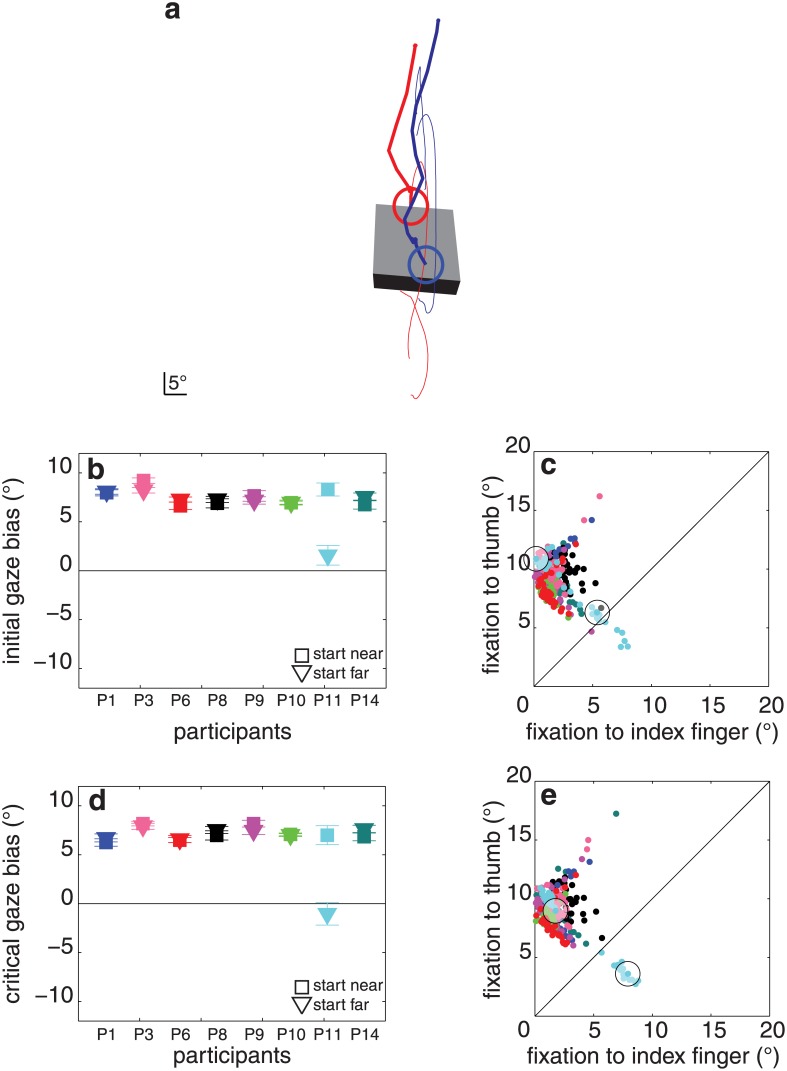
Results of experiment 4. (**a**) Two trials of a single participant (P11), one with the hand starting from the *far* position (blue trajectories) and one with it starting from the *near* position (red trajectories). (**b**) Initial gaze bias for each starting position and participant. **(c)** Distance between the first fixation and each of the two contact points for each of the 324 trials. (**d, e**) Same as **b**, **c** for the critical fixation. Details as in Fig 3.

Fig 9a shows two trials of a single participant, each with the hand starting its movement from a different position. This participant brought her gaze down to near where her thumb made contact with the object when starting from the *far* position (blue trajectories), and close to where her index finger made contact with the object when starting from the *near* position (red trajectories), as we proposed that might happen. However, this was not a general tendency.

The initial gaze bias was 7.3 ± 1.5° (t_7_ = 19, p < 0.001; Fig 9b and 9c). The critical gaze bias was 6.7 ± 1.7° (t_7_ = 12, p < 0.001; Fig 9d and 9e). The initial hand position did not influence the critical gaze bias (F_1,7_ = 0.5, p = 0.5; Fig 9d). There was also no effect of the initial hand position on contact asynchrony (F_1,7_ = 0.05, p = 0.7) which was 12 ± 11 ms. No significant correlation was found between contact asynchrony and the critical gaze bias (r = 0.12, p = 0.7). The median maximal distance between fixations was 1.4 ± 0.8°. The average final grip size was 9.9 ± 1.1°.

### Discussion

In this experiment we tested whether the gaze bias depends on the curvature of the digit’s movement towards its contact point. One participant (P11) did tend to look near where her thumb made contact with the object when starting the movement from the far position, as we had anticipated, but otherwise neither the initial nor the critical gaze bias was influenced by the initial hand position. This shows that the bias towards the index finger’s contact point is not actually a bias towards the contact point of the digit that curves around the object’s surface.

## Experiment 5—Increasing Required Accuracy

**Fig 10:**
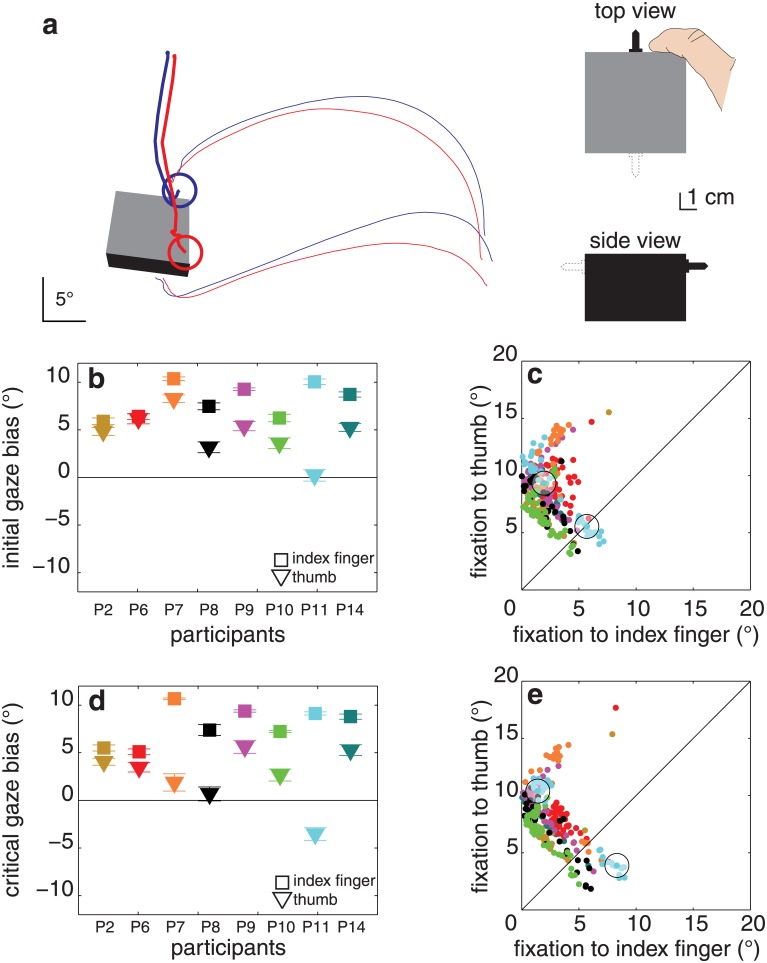
Results of experiment 5. (**a**) Two trials of a single participant (P11), one with the protrusion attached to the index finger’s contact surface (blue trajectories) and one with the protrusion attached to the thumb’s contact surface (red trajectories). The drawings on the right show the placement of the protrusion (both possible positions are shown but on each trial there was only one protrusion, here the highlighted one at the index finger’s side). The drawn digit (approximately drawn to scale) gives an impression of how accurate the placement of the digit had to be to avoid the protrusion (when present). (**b**) Initial gaze bias for each protrusion position and participant. **(c)** Distance between the first fixation and each of the two contact points for each of the 316 trials. (**d, e**) Same as **b**, **c** for the critical fixation. Details as in Fig 3.

A manipulation that will more obviously influence the actual grasp than does placing a sphere at the edge of a surface (experiment 3) or choosing initial hand positions that force one of the digits to curve around the target object (experiment 4), is to attach a protrusion near the contact point on one of the surfaces. Changing the object’s shape, so that more accuracy is required by one digit than by the other, has been shown to give rise to small shifts in gaze [18]. In this experiment, we attached a protrusion on one side of the block (Fig 10a), thereby constraining the placement of either our participants’ thumb or index finger. With a protrusion at the side of the thumb, our participants might look closer to their thumb’s contact point to make sure not to hit the protrusion. All other details were the same as in experiment 1, except that we only used one orientation of the block (90° relative to the frontal plane).

### Participants

Eight participants (P2, P6—P11, P14) took part in this experiment. Of the 352 trials (8 participants; 2 protrusion positions; 22 replications), 36 had to be discarded, mainly because markers on the digits were occluded, resulting in 316 trials for further analysis.

### Results

Fig 10a shows two trials of a single participant, with the protrusion at different sides of the object. The participant brought her gaze to near where her index finger later made contact with the object when the protrusion was at the side at which her index finger made contact with the object (blue thick trajectory), whereas she brought her gaze close to where her thumb later made contact with the object when the protrusion was at the side at which her thumb made contact with the object (red thick trajectory). Again, this was not a general tendency.

The initial gaze bias was 6.3 ± 1.7° (t_7_ = 11.3, p < 0.001; Fig 10b and 10c). The critical gaze bias was 5.4 ± 2.3° (t_7_ = 8.3, p < 0.001; Fig 10d and 10e). The protrusion’s position did influence the critical gaze bias (F_1,7_ = 17.03, p < 0.005; Fig 10d): the critical gaze bias was 7.9 ± 1.9° when the protrusion was at the index finger’s side, and only 2.9 ± 2.7° when the protrusion was at the thumb’s side. Note that the average critical gaze bias was still positive (towards the index finger) when the protrusion was at the thumb’s side. No effect of the protrusion’s position was found on contact asynchrony (F_1,7_ = 1.9, p = 0.6; average: 12 ± 16 ms). No significant correlation was found between contact asynchrony and the critical gaze bias (r = 0.24, p = 0.5). The median maximal distance between fixations was 2.1 ± 1.2°. The average final grip size was 9.4 ± 1.2°.

### Discussion

Imposing constraints on the digits’ contact points led to slight changes in both the initial and critical gaze bias. Although fixations shifted closer to the thumb’s contact point when the thumb had to avoid the protrusion, the average gaze bias remained positive. This shows how strongly people prefer to fixate near their index finger’s contact point. Thus the required accuracy in placing the digit influences gaze, but it is not responsible for the bias.

## Experiment 6—Thumb and Little Finger

Even when a protrusion was attached near the position at which the thumb would normally contact the object, our participants still looked closer to their index finger’s contact point. The protrusion did influence gaze, but for most participants the effect was quite modest, although the protrusion clearly constrained the movement of one of the digits. Thus, perhaps it is not the actual constraints that matter, but the fact that the index finger normally requires more visual guidance in daily life, because it is usually the digit that moves around the object that is to be grasped, so people may just look at its contact position out of habit. To examine whether people look near the index finger’s contact point out of habit, we asked participants to grasp the oriented block (configurations as in experiment 1) between their thumb and little finger. If actual constraints are critical, we would expect participants to look even closer to their little finger’s contact point than they do for the index finger, because it is less common to grasp objects in this manner, so doing so is likely to require more visual control. If the eyes are simply directed to the index finger’s contact point out of habit, it is not clear what they will do if the index finger is not involved in the grasp. For this experiment, looking closer to the little finger’s contact point and making contact first with the little finger are considered to be positive biases. All further details in this experiment were identical to those in experiment 1.

### Participants

Seven participants (P5—P7, P9, P11, P13, P14) took part in this experiment. Of the 462 trials (7 participants; 3 object orientations; 22 replications), 77 had to be discarded, mainly because of equipment failure and because of uncertainty about the contact times, resulting in 385 trials for further analysis.

### Results

**Fig 11:**
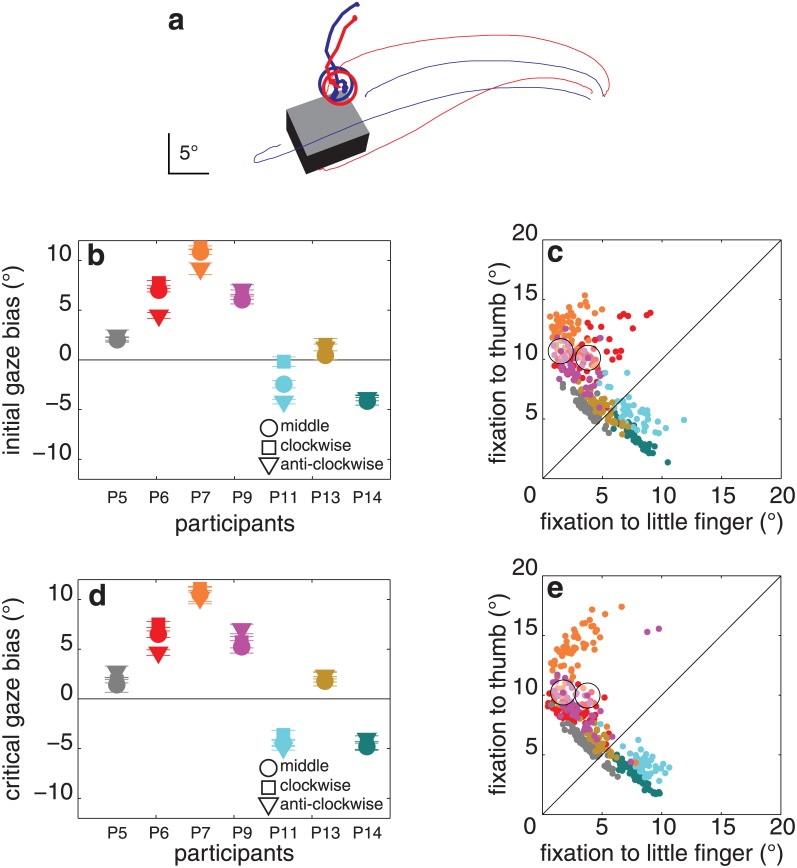
Results of experiment 6. (**a**) Two trials of a single participant (P9) for one object orientation and the object grasped by different pairs of surfaces (blue and red lines). (**b**) Initial gaze bias for each object orientation and participant. **(c)** Distance between the first fixation and each of the two contact points for each of the 385 trials. (**d, e**) Same as **b**, **c** for the critical fixation. Details as in Fig 3.

Fig 11a shows two trials of a single participant for the middle object orientation. In both trials, the participant fixated close to where the little finger made contact with the object (blue and red thick trajectories).

The participants’ initial gaze bias was not significantly different from zero: 3.9 ± 4.0° (t_6_ = 1.5, p = 0.1; Fig 11b and 11c). The participants’ critical gaze bias was not significantly different from zero either: 2.8 ± 4.8° (t_6_ = 1.2, p = 0.2; Fig 11d and 11e). The object’s orientation did not influence the critical gaze bias (F_2,12_ = 1.9, p = 0.2; Fig 11d). There was no effect of object orientation on contact asynchrony (F_2,12_ = 3.8, p = 0.6; average: 11 ± 18 ms). No significant correlation was found between contact asynchrony and the critical gaze bias (r = 0.68, p = 0.1). The median maximal distance between fixations was 1.9 ± 0.9°. The average final grip size was 10.9 ± 1.1°.

### Discussion

In this experiment, the gaze bias differed considerably across participants, which could mean that people fixate near the index finger’s contact point out of habit, leading to variability when the index finger no longer has a contact point. However, we certainly cannot be confident about this because some participants might look closer to their thumb than they would when grasping with the index finger (compare P11 in Figs 11b–11d and 3b–3d) because their own hand occludes the top of the object when grasping it with the little finger, while others might look closer to their little finger’s anticipated contact point (compare P7 in Figs 11b–11d and 3b–3d) because they expect to require more visual guidance for grasping with a less commonly used digit.

If people fixate certain positions out of habit, they might even be biased to look close to where their index finger would normally land, rather than to where they plan for it to land on the trial in question, in which case replacing the index finger by the little finger should not change much (other than as a result of the above-mentioned occlusion). Note that although the variability between the participants’ biases is larger in this experiment than in the former ones, the distance between fixations during a trial and the variability across trials for individual participants were not larger, so participants were consistently doing different things, not just shifting their gaze around to learn where they can best look during this unusual way of grasping.

## Experiment 7—Two Index Fingers

In the previous experiment we found that some people looked near the contact point of their little finger, but the tendency was less consistent than when using the index finger. To further examine whether people fixate near their index finger’s contact point out of habit, we asked participants to reach and grasp a block between the index fingers of both their hands. The block could have one of three different orientations (90°, 70° or 110° relative to the participant’s frontal plane; we will refer to these orientations as *middle*, *clockwise* and *anti-clockwise*, respectively). They started with their hands close to their body, as in the *near* condition of experiment 4. For stability, they held their hands together with the digits interleaved [35]. If people look near where their index finger is heading because this digit needs more visual control, then in this experiment we would expect our right-handed participants to look near the contact point of their left index finger, following the same reasoning as for the little finger in the previous experiment. If they look where the index finger will land out of habit, it is presumably the contact point of the right index finger that matters. We consider looking closer to the right index finger and first making contact with the right index finger to be positive biases. All other details were the same as in experiment 1.

### Participants

Seven participants (P1, P4, P6, P7, P9—P11) took part in this experiment. Of the 462 trials (7 participants; 3 object orientations; 22 replications), 56 had to be discarded, mainly because of missing markers, resulting in 406 trials for further analysis.

### Results

**Fig 12:**
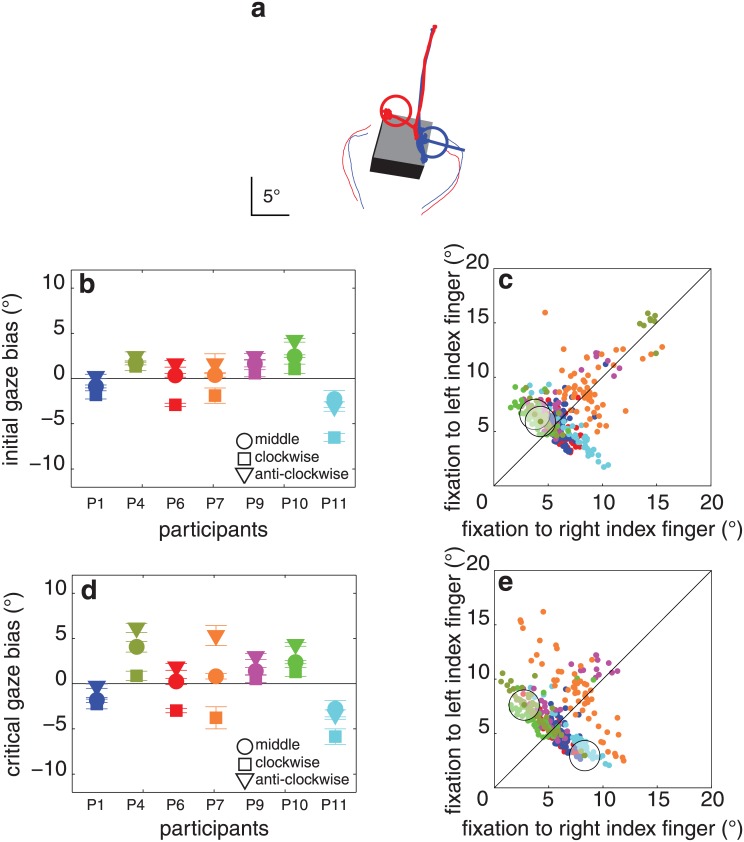
Results of experiment 7. (**a**) Two trials of a single participant (P4) for the clockwise object orientation, one with the gaze at the time of contact close to the right index finger’s contact point (blue) and the other with the gaze at that time close to the left index finger’s contact point (red). (**b**) Initial gaze bias for each object orientation and participant. **(c)** Distance between the first fixation and each of the two contact points for each of the 406 trials. (**d, e**) Same as **b**, **c** for the critical fixation. Details as in Fig 3.

Fig 12a shows two trials of a single participant for the middle object orientation. In one of these trials the participant fixated close to where the right index finger made contact with the object (blue thick trajectory). In the other she initially looked close to where her right index finger later made contact with the object and then shifted her gaze close to where her left index finger later made contact with the object (red thick trajectory).

Participants neither showed a significant initial gaze bias (0.02 ± 1.7°, t_6_ = 0.3, p = 0.7; Fig 12b and 12c) nor a significant critical gaze bias (0.3 ± 2.2°, t_6_ = 0.6, p = 0.5; Fig 12d and 12e). Interestingly, the object’s orientation did influence the critical gaze bias (F_2,12_ = 11.7, p = 0.002; Fig 12d): it was negative for the clockwise object orientation (-1.5 ± 1.7°) and positive for the anti-clockwise (1.8 ± 2.7°) and middle object orientations (0.7 ± 2.1°). This means that participants looked nearer to the contact point of the digit that needed to curve around the object’s corner that constrained the movement towards the contact point. There was also an effect of object orientation on contact asynchrony (F_2,12_ = 6.3, p = 0.01): it was 3 ± 10 ms for the middle orientation, 18 ± 12 ms for the clockwise orientation, and -10 ± 15 ms for the anti-clockwise object orientation. This means that the digit that made first contact with the object was the one that did not need to curve around the object’s corner to reach its contact point as in [26]. No significant correlation was found between contact asynchrony and the critical gaze bias (r = 0.18, p = 0.7). The median maximal distance between fixations was 2.1 ± 1.1°. The average final grip size was 10.9 ± 1.8°.

### Discussion

Our participants, who were all right-handed, did not have a systematic gaze bias towards the right index finger’s contact point, so the bias that we found in the other experiments cannot simply be due to participants looking where the right index finger is going out of habit. We found that our participants’ critical gaze was biased towards the contact point of the index finger that had to curve around the object, so it seems that avoiding possible collisions with the corners of the object might determine where one looks to some extent (contrary to our conclusions from experiment 4) in some conditions.

## General Discussion

In this study, we aimed at better understanding why people generally look near their index finger’s contact point when grasping an object. We found that fixations depended on where our participants were looking before they started the grasping movement, on the presence of protrusions, and on the orientation of the object when grasping with the index fingers of both hands. Fixations did not depend on the other factors that we examined, including the position at which the grasping movement itself started.

In our first experiment, we asked participants to grasp a block that was placed on a table in front of them in various orientations. The hand was initially to the right of the object. In this way we tried to minimize the differences in the constraints imposed on the two digits, while maintaining a natural grasping movement. In accordance with previous studies ([17–20]; but see [26]), we found that our participants generally fixated near the contact point of their index finger. In some object orientations, participants grasped the block by different pairs of surfaces on different trials. Their fixations often shifted in accordance with the grip that was used (Fig 4; also see Fig 6), so they were not simply looking at the top of the object. The first fixations were clearly near the index finger’s contact point. Occasionally, participants shifted their gaze closer to their thumb’s contact point by the critical moment of the grasp (compare for instance P7 in Fig 3c and 3e), but fixations near the thumb’s contact point were quite rare (only a few points below the diagonal of the results figures in the experiments involving the thumb).

The tendency that we found in experiment 1 to fixate near the contact point of the index finger was not just due to the starting position of the gaze being closer to that contact point. In experiment 2, we moved the starting position of the gaze to the side of the thumb’s anticipated contact point. Although the initial and critical gaze biases were reduced in experiment 2, they did not change sign. The critical fixations of all but one participant (P11) were still clearly closer to the index finger’s contact point than to the thumb’s contact point. In experiment 2, the bias to look near the index finger’s contact point appeared to be less strong for first than for later fixations (as previously reported: [17,18]). Since we found the opposite pattern in experiment 1, we propose that the starting position of the eyes primarily influences the first fixation, probably because making shorter saccades means that one obtains information about the object sooner.

We also confirmed (experiment 4) that the tendency of the index finger to move along a more curved trajectory is not responsible for critical fixations being near the index finger’s contact point [19], despite the edge that the digit had to curve around forming an obstacle on the way to the contact point [10]. Only one of our participants (P11) shifted her critical fixations to near the contact point of the digit that had to curve over the object. The others fixated near their index finger’s contact point, irrespective of the hand’s initial position. Nevertheless, in experiment 7, we did see a tendency to direct gaze towards the contact point of the digit that had to move around an edge of the object. Since the difference in the extent to which the digits have to follow a curved path is much larger in experiment 4 than in experiment 7, it is unlikely to be the fact that the digit has to curve around an edge that is critical. A more likely option is the fact that the surface of the contact point is out of sight. In experiments 1–5, the index finger always moved to the surface that was facing away from the participant, so the surface at which it would contact the object was always out of sight. In experiment 7, the surface that was out of sight depended on the object’s orientation, as did the gaze (there was a bias to look towards the contact point on the surface that was facing away from the participant). This dependence of gaze bias on visibility would not explain why people look at their index finger when grasping a flat object attached to a more or less frontal plane [17–19], but it could explain why people tend to look in the direction of their index finger when grasping opaque objects off a horizontal surface.

It is somewhat surprising that the subtle differences in object orientation in experiment 7 influence gaze as much as does placing protrusions at the centre of the grasping surfaces in experiment 5. Perhaps the possibility of something obstructing one’s movements to a position that one cannot see (protrusion near the index finger’s contact point in experiment 5) attracts gaze more than does the presence of an object at a position that one can see (protrusion near the thumb’s contact point). This could explain why only one participant (P11) fixated nearer to her thumb’s contact point when the protrusion was on the thumb’s side in experiment 5, whereas the other participants fixated closer to their index finger’s anticipated contact point (despite the additional spatial requirements at the thumb). It is also consistent with gaze sometimes being directed towards occluded parts of objects in previous studies [17]. This might even explain why gaze is directed to the thumb’s contact point when the object is at eye height [26], because with the index finger’s contact point completely behind the object it is evident that looking closer to the index finger cannot help one see potential obstacles on the index finger’s trajectory.

Another idea that has been proposed to explain systematic fixations towards the index finger’s contact point is that the index finger generally contacts the object earlier than the thumb [19]. Although we failed to make participants systematically first contact the object with their thumb in experiment 3, we presented an analysis of the data that examines this hypothesis in all experiments. When correlating the participants’ average contact asynchronies with their average critical gaze biases we found no significant correlations in any of the experiments. The consistent absence of a clear correlation between the critical gaze bias and the contact asynchrony suggests that it is not very likely that this is a critical factor in determining where one fixates when grasping.

On average, the index finger contacted the object about 15 ms earlier than the thumb in our first five experiments, which is much less than the difference reported in some earlier studies (about 55 ms and 84 ms reported in [19] and [18], respectively). The difference might arise from the differences between the objects that were to be grasped, or the differences between how they were placed, but it might also arise from the method used to determine the moment of contact. Previous authors determined the contact asynchrony as the difference between when each digit’s velocity dropped below a certain threshold. We used a more sophisticated method that captures the most likely moment of contact for each digit by combining multiple sources of information (see *2*.*5 Data analysis*; [28]). There is very little asynchrony in the moment of initial contact (as measured using force sensors); the asynchrony arises due to a longer deceleration for the thumb [33]. It is therefore likely that the differences in the extent of the asynchrony between studies are due to differences in the data analysis rather than differences between objects or the way they were grasped.

Another possible reason for the systematic bias to fixate near the index finger’s contact point is that doing so may usually be the optimal thing to do when grasping objects in daily tasks. If the bias were directly related to where the index finger is heading, we would not have expected participants to fixate near their little finger’s contact point in experiment 6. That people’s first and critical fixations were generally near their little finger’s contact point, although some participants did fixate near their thumb’s contact point, suggests that the bias to fixate the index finger’s contact point in the previous experiments does not just arise out of habit. However, the larger variability in the fixation positions across participants in experiment 6 could arise from participants fixating the surface of the object near where their index finger will be at the moment of the grasp. The position of the index finger was not recorded in experiment 6, so we cannot completely exclude this possibility. We find it unlikely, though, because we do not have any reason to expect participants to fixate a region of the object near a digit that does not actually make contact with the object.

Additional evidence against looking at the index finger’s contact point out of habit is provided by experiment 7. The participants’ first and critical fixations were neither systematically closer to the left nor closer to the right index finger’s contact point, suggesting that fixations neither depend on how often a digit is used nor on how much control it requires. Instead, participants in experiment 7 fixated nearer the surface that was oriented away from themselves. Their fingers first made contact with the surface that was oriented towards themselves. Thus, their critical fixations were not closer to the digit that contacted the object first. It therefore appears to be the position that is on the far, occluded side of the object, rather than the index finger’s contact point, that attracts gaze. We propose that there is a tendency to look closer to the surface that one does not see because the precise contact is less predictable at that surface; it is more likely that one will encounter unexpected collisions with occluded objects behind the object that one is grasping, or that one will encounter unexpected dents or protrusions of the surface of the object itself.

One might have expected our participants to shift their gaze sequentially between both anticipated contact points and direct their gaze to the most important position at the critical moment. Our participants did not do this: the overall average maximal shift in gaze within a single trial (after the first saccade; in any direction) was 2.6 ± 0.6°, which is only a small fraction of the average final grip size (10.6 ± 1.3° between the markers on the nails; possibly slightly less between the digits’ contact points) and is clearly smaller than the average critical fixation bias (about 5°).

Although the selection of contact points is critical when planning and executing a grasping movement, we here found that looking directly at both these points does not seem to be particularly important. Most importantly, we show that the strong bias to fixate near the index finger’s contact point, even in head-free grasping, cannot be fully explained by any of the factors that have been proposed and that we examined. What we do show is that where one is looking before one starts the grasping movement, what the required accuracy is at the contact area, and whether the digit’s contact point is visible or not, all bias where one fixates.
